# Identification of vitamin B_12_ producing bacteria based on the presence of *bluB*/*cobT2* homologues

**DOI:** 10.1007/s10529-023-03362-2

**Published:** 2023-03-13

**Authors:** Darya Dudko, Sofia Milker, Dirk Holtmann, Markus Buchhaupt

**Affiliations:** 1 Microbial Biotechnology, DECHEMA Research Institute, Theodor-Heuss-Allee 25, 60486 Frankfurt am Main, Germany; 2 Faculty Biology and Chemistry, Justus-Liebig-Universität Gießen, Ludwigstraße 23, 35390 Gießen, Germany; 3 Institute of Bioprocess Engineering and Pharmaceutical Technology, University of Applied Sciences Mittelhessen, Wiesenstrasse 14, 35390 Gießen, Germany

**Keywords:** BluB/CobT2, Cobalamin, LC–MS/MS, Method validation, Pseudovitamin B_12_, *Terrabacter* sp., Vitamin B_12_

## Abstract

**Objectives:**

The objective of the study was to develop a strategy for the identification of new vitamin B_12_-producing species and to characterize their production capability using a fast and sensitive LC–MS/MS method developed in this study.

**Results:**

Searching for homologues of the *bluB*/*cobT2* fusion gene known to be responsible for the production of the active vitamin B_12_ form in *P. freudenreichii* was shown to be a successful strategy for the identification of new vitamin B_12_-producing strains. The analysis of the identified strains via LC–MS/MS showed the ability of *Terrabacter* sp. DSM102553, *Yimella lutea* DSM19828 and *Calidifontibacter indicus* DSM22967 to produce the active form of vitamin B_12_. Further analysis of vitamin B_12_ production capability of *Terrabacter* sp. DSM102553 in M9 minimal medium and peptone-based media revealed that the highest yield of 2.65 µg of vitamin B_12_ per g dry cell weight was obtained in M9 medium.

**Conclusions:**

The proposed strategy enabled identification of *Terrabacter* sp. DSM102553, whose relatively high yields obtained in the minimal medium open new perspectives for the possible application of the strain for biotechnological vitamin B_12_ production.

**Supplementary Information:**

The online version contains supplementary material available at 10.1007/s10529-023-03362-2.

## Introduction

Vegetarian and vegan diets have gained popularity as alternative ways of nutrition in large parts of the world in recent years. However, vegans are at a particular risk of vitamin B_12_ deficiency since this vital nutrient naturally originates only from foods of animal origin or fortified foods fermented with bacteria (Chamlagain et al. 2017).

**Fig. 1:**
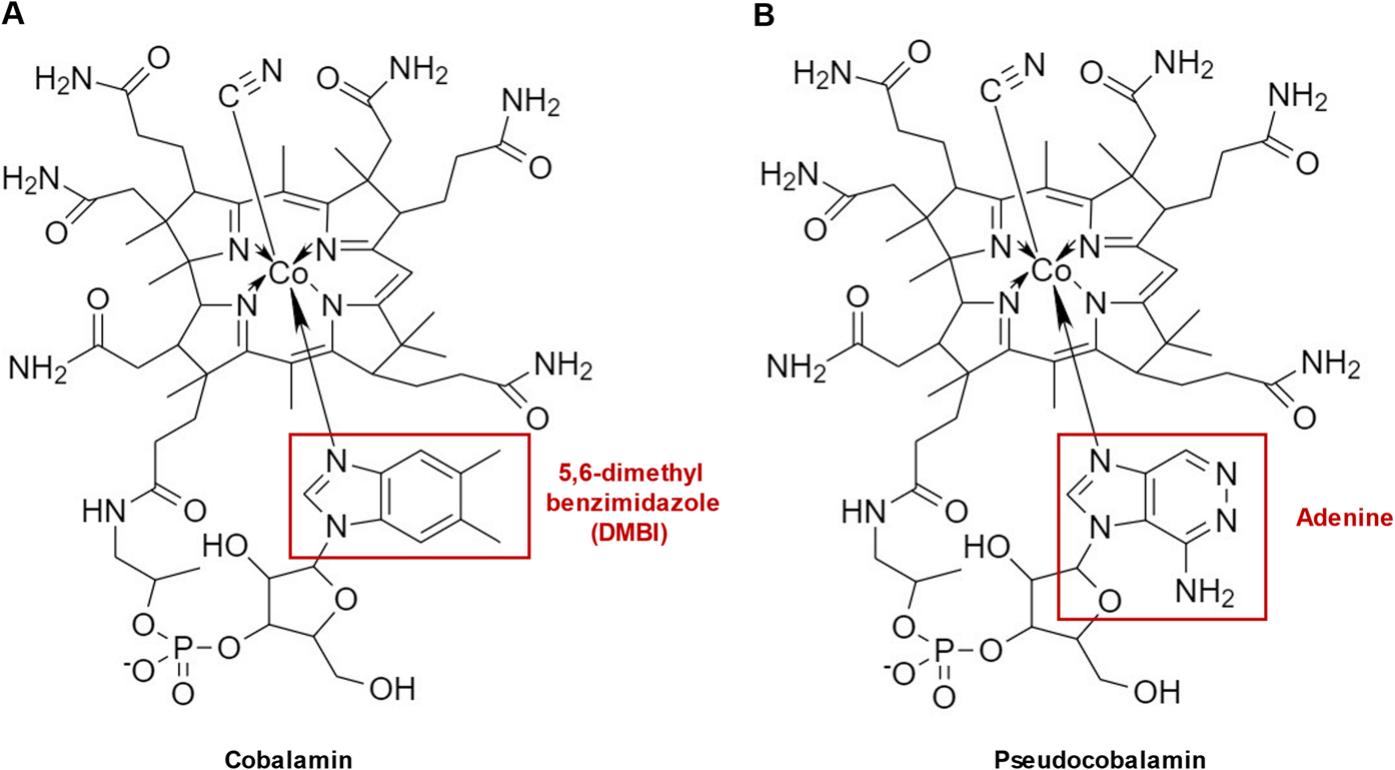
Chemical structures of active vitamin B_12_ (**A**) and pseudovitamin B_12_ analogue (**B**): The differences in the lower ligand structure leading to the characteristic fragmentation patterns are marked in red

Vitamin B_12_ (cobalamin) plays a crucial role for the human body since it is used as a cofactor for two important enzymes, methylmalonyl-CoA mutase and methionine synthase (Martens et al. 2002). Methionine synthase is known to be involved in DNA synthesis (Weyden et al. 1973), while methylmalonyl-CoA mutase plays an important role in branched amino acid and odd-chain fatty acid metabolism (Takahashi-Iñiguez et al. 2012). Despite such essential requirement, the human metabolism is not capable of vitamin B_12_ production, which results in a need for products enriched with vitamin B_12_. Due to the very complex chemical synthesis of vitamin B_12_, microbial biosynthesis is applied for its commercial production (Martens et al. 2002). It is known for a long time that several bacteria belonging to different genera are able to synthesize vitamin B_12_ (Perlman 1959), while other reports have also demonstrated high amounts of vitamin B_12_ produced by cyanobacteria of the genus *Spirulina* (Berg et al. 1988). Nevertheless, the commercially available *Spirulina* supplements mainly contain a corrinoid later identified as pseudovitamin B_12_ (Watanabe et al. 1999, 2001), a vitamin B_12_ analogue which cannot be utilized as cofactor by human enzymes. The structures of the vitamin B_12_ forms active in humans and the pseudovitamin B_12_ are very similar (Fig. 1).

The lower ligand of the cobalamin molecule is represented by 5,6-dimethylbenzimidazole (DMBI) in the active forms of vitamin B_12_ or by adenine in pseudovitamin B_12_, respectively (Watanabe 2007). Due to the biological inactivity of pseudovitamin B_12_ in humans, determination of the type of the synthesized cobalamin compound during the microbiological production is very important (Chamlagain et al. 2017).

Besides *Pseudomonas denitrificans, Propionibacterium freudenreichii* (previously designated as *P. shermanii*) is used for the industrial production of vitamin B_12_ (Martens et al. 2002) due to the ability to nearly exclusively synthesize the active vitamin B_12_ (Deptula et al. 2017). The high levels of the produced active vitamin B_12_ in *P. freudenreichii* were shown to be the result of the BluB/CobT2 fusion enzyme activity in this strain (Deptula et al. 2015). The study demonstrated that BluB enzyme is responsible for the formation of the lower ligand DMBI, while CobT2 facilitates the activation and the subsequent incorporation of DMBI into the cobalamin molecule.

Based on the data reported by Deptula et al. 2015, we aimed to identify new, previously not described cobalamin-producing species by evaluation of bacterial strains containing a gene encoding the BluB/CobT2 fusion enzyme. In addition, a sensitive and reliable LC–MS/MS method for the identification and quantification of vitamin B_12_ was used to screen the identified candidates for cobalamin (here termed as the active form of vitamin B_12_).

## Materials and methods

### ***Development of the method for vitamin B***_***12***_*** analysis***

The sample analysis was performed with a triple quadrupole LCMS-8045 (Shimadzu, Germany) and the Lab Solutions Analysis Software (Shimadzu, Germany) was used for data acquisition and analysis. A Luna® Omega 3 µm PS C18 100 Å Column (Phenomenex, Germany) was operated with 0.1% formic acid in water (solvent A) and 0.1% formic acid in acetonitrile (solvent B). All used solvents were LC/MS grade (Carl Roth, Germany). The following LC time program was used: 0–3 min 18%–32% B, 3–3.1 min 32%–95% B, 3.1–4.1 min 95% B, 4.1–4.3 min 95%–18% B, 4.3–7 min 18% B. The flow rate was 0.4 ml min^−1^ and the column temperature was maintained at 40 °C. The MS analysis was carried out in positive ion mode using electrospray ionization (ESI) under following parameters: nebulizing gas flow 3 l min^−1^, drying gas flow 10 l min^−1^, interface temperature 300 °C, desolvation line temperature 250 °C and heat block temperature 400 °C. The mass spectrometer was run in multiple reaction monitoring (MRM) mode, method parameters (collision energies, dwell times and exact m/z values) were optimized for the double-charged cyanocobalamin ion [M + 2H]^2+^ with 678.40 m/z and for the double-charged pseudocobalamin ion [M + 2H]^2+^ with 672.75 m/z with the software. The injection volume was 1 µl and the quantification of vitamin B_12_ in the samples was performed using a calibration curve obtained from a set of cyanocobalamin standards (Merck, Germany). Since pseudovitamin B_12_ is not commercially available as analytical standard, the cell extract of *L. reuteri* containing pseudovitamin B_12_ was used as reference material.

### Method validation

The instrumental detection limit (LOD) was determined as the lowest concentration of cyanocobalamin standard corresponding to the first peak that can be integrated and distinguished from zero using the signal-to-noise (S/N) approach (S/N ratio ≥ 3). The limit of quantification (LOQ) was defined as the lowest concentration of cyanocobalamin that can be determined with an acceptable repeatability (relative standard deviation (RSD) between the samples under 10%) using the signal-to-noise (S/N) approach (S/N ratio ≥ 10). The linearity of the method was estimated over the concentration range of 20–2000 nM of CNCbl, a set of 10 concentrations of the standard solution was used, each solution was injected in triplicate. The determination coefficient R^2^ was calculated to estimate the linearity. To evaluate the selectivity of the method, the chromatograms of vitamin B_12_-free acetate buffer and *E. coli* DSM18039 cell extracts were compared with the chromatograms the acetate buffer and *E. coli* DSM18039 cell extracts spiked with cyanocobalamin standard.

Commercially available vitamin B_12_ supplements were used for assessing the accuracy of the method. Five different samples containing various amounts of cyanocobalamin were spiked with a known amount of cyanocobalamin standard and the accuracy was estimated through the recovery rates which were calculated as described by Campos-Gimnez et al. 2008:

$$ \frac{{{\text{Cs}} - {\text{Cn}}}}{{{\text{Ca}}}} \times 100 $$

where Cs is the concentration of vitamin B_12_ found in the spiked sample, Cn is the concentration of vitamin B_12_ in the native sample, and Ca is the concentration of cyanocobalamin added in the spiked sample.

The within-day repeatability was evaluated in the range of 100–1000 nM by analysing five different samples, each solution was injected in triplicate, while the intermediate reproducibility (between-day) was determined by analysing a calibration row of five different samples in the range of 100–1000 nM during three consecutive days.

### Sequence analysis

The BluB/CobT2 protein sequence [GenBank: CBL56167.1] from *P. freudenreichii* was used for the identification of bacterial homologues by means of BLAST (National Center for Biotechnology Information). The BLASTp tool was applied for the search in the protein sequence database excluding the genus *Propionibacterium* (taxid:1743), the maximum number of aligned sequences was set to 1000.

### Strains and cultivation conditions

*Lactobacillus reuteri *DSM20016 used for pseudovitamin B_12_ purification, *E. coli* DSM18039 and all strains identified in this work as candidates for vitamin B_12_ production were obtained from DSMZ (German Collection of Microorganisms and Cell Cultures, Braunschweig, Germany). The cultivation of microorganisms was performed in the media recommended by DSMZ for every specific strain: *Terrabacter* sp. DSM102553 and *Terrabacter* sp. DSM102554 in medium 513 (PP), *Calidifontibacter indicus* DSM22967 and *Raineyella antarctica* DSM100494 in medium 92, *Yimella lutea* DSM19828 in medium 65, *Blastococcus* sp. DSM44272 in medium 714, *E. coli* DSM18039 in medium 381 and *Lactobacillus reuteri* DSM20016 in medium 11, respectively. A specific M9 minimal medium (Rüdiger and Schwab 2019) and medium 513 with the doubled amount of all components (2xPP) were also used in the growth experiments with *Terrabacter sp*. DSM102553. 50 ml cultures were grown in 300 ml shaking flasks at 30 °C under aeration and shaking conditions.

Anaerobic cultivation was applied for the production of pseudovitamin B_12_ with *L. reuteri* DSM20016. Briefly, the pre-cultures were inoculated from the glycerol culture stocks (− 80 °C) in 50 mL medium and incubated in septum flasks anaerobically for 24 h at 30 °C.

### Cultivation of bacterial strains in BioLector microbioreactor

The pre-cultures of the candidate strains were grown in respective media for 48 h and used for the inoculation of 1 mL medium at the starting OD_600_ of 0.1. The cultivation was carried out in a BioLector® MB system (m2p-labs, Germany) in MTP-48 FlowerPlates® without pH optodes at 30 °C, 1000 rpm and 95% humidity. The growth of the cultures was monitored online by scattered light signal measurement with an excitation wavelength of 620 nm.

### ***Vitamin B***_***12***_*** and pseudovitamin B***_***12***_*** extraction, purification and analysis***

For the cobalamin extraction 25 ml of the cultures of the identified candidate strains were harvested by centrifugation at 315×*g* for 30 min after 5 days of cultivation, while the complete biomass of *L. reuteri* DSM20016 obtained after 24 h of cultivation was applied for the purification of pseudocobalamin. The cell pellets were resuspended in 10 ml of acetate buffer (4.1 g sodium acetate l^−1^ adjusted to pH 4.5 with acetic acid) containing 100 µl of 1% KCN. After incubation in a water bath at 98 °C for 30 min the samples were cooled on ice for 30 min and centrifuged again. Vitamin B_12_ and pseudocobalamin were purified from the obtained supernatants using BAKERBOND spe™ C18 columns JB7020-03 (J. T. Baker) according to the manufacturer’s instructions. The extracts were then syringe sterile filtered (0.2 µm), dried at 60 °C under vacuum, resuspended in 100 µl of deionized H_2_O and then used directly for the vitamin B_12_ analysis with the above described LC–MS/MS method.

## Results and discussion

### LC–MS/MS method development and validation

Since the widely used microbiological assay is not able to differentiate between active forms of vitamin B_12_ and its analogues (Chamlagain et al. 2015), the first objective of this study was to develop a suitable LC–MS/MS method for vitamin B_12_ analysis. The parent and fragment ion masses of m/z 678.40, m/z 146.95 and m/z 359.10, which were identified in the spectrum of the active cobalamin have already been demonstrated in previous reports on LC–MS/MS-based methods for vitamin B_12_ determination (Lu et al. 2008; Luo et al. 2006; Schwertner et al. 2012; Szterk et al. 2012; Lee et al. 2015; Zironi et al. 2014). The demonstrated LOQ was comparable with that of the method described by Yi et al. 2012 which was also based on the tandem triple quadrupole mass spectrometry in MRM mode. In comparison to previously described LC–MS/MS methods by Schwertner et al. 2012, Lee et al. 2015, Szterk et al. 2012, the method reported in this study provides a faster analytical procedure and can, therefore, enable a higher sample throughput. Moreover, only few micrograms of cell material were sufficient for the successful B_12_ analysis with the developed method, while earlier reported LCMS/MS-based methods applied 15–30 g of sample material for vitamin B_12_ purification for subsequent analysis (Szterk et al. 2012; Luo et al. 2006).

The method was linear within the tested range over 10 calibration levels with R^2^ = 0.9997. The selectivity of the method was evaluated by comparing the chromatograms of the buffer and vitamin B_12_-free *E. coli* MG1655 cell matrix blanks with the same samples spiked with cyanocobalamin standard. The comparison of the blank samples with the spiked samples showed a high selectivity of the method, since no peaks with the fragmentation pattern and the retention time corresponding to cyanocobalamin were detected in the controls whereas all spiked samples demonstrated cobalamin peaks. Although an unidentified peak was observed in the chromatogram of the cell extract of *E. coli* DSM18039 (Supplementary Fig. 1), the fragment signals with m/z 456.75 and m/z 359.10 characteristic for cyanocobalamin were not detected. This allows to exclude that the observed peak corresponds to CNCbl and is rather evidence for a substance with the same precursor ion mass as cyanocobalamin but another chemical structure.

The accuracy of the method was determined through recovery by spiking of five different commercially available vitamin B_12_ supplements with known amounts of CNCbl standard. The average recovery was estimated in technical triplicates by determination of the peak areas of the samples before and after spiking. The method demonstrated high accuracy, since the average recovery from spiked samples was between 94.4% and 103.4% (Supplementary Table 1), which was comparable with the previously reported LC–MS/MS-based methods for vitamin B_12_ determination (Lu et al. 2008; Luo et al. 2006).

The within-day repeatability was evaluated in the range of 100–1000 nM by analyzing five different samples, measured as technical triplicates. The relative standard deviation in the repeatability experiment was less than 4%. The intermediate precision (between-day or day-by-day) was determined by analyzing calibration series of five different samples in the range of 100–1000 nM during three consecutive days. The RSD in the intermediate reproducibility was below 4%.

### Identification of strains containing a bluB/cobT2 fusion gene

Activity of the fusion enzyme BluB/CobT2, which produces and directly activates DMBI, is the main mechanism responsible for the high levels of active vitamin B_12_ in *P. freudenreichii* cells, which has been shown in an earlier study (Deptula et al. 2015). Furthermore, the metabolite channeling feature of fusion enzymes can contribute to high flux through a biosynthetic pathway by protection of reactive intermediates and provision of high local substrate concentration (Kummer et al. 2021). Therefore, we aimed at the investigation of microorganisms with a *bluB*/*cobT2* fusion gene for their vitamin B_12_ production capabilities.

The results of a respective BLAST search were analyzed and the sequences showing an identity of more than 50% to the BluB/CobT sequence from *P. freudenreichii* were chosen. From the resulting list of 338 sequences identified at the time of the investigation, we randomly selected six non-pathogenic organisms (risk group 1) for further analysis (Supplementary Table 2). The identified candidate strains *C. indicus* DSM22967, *R. antarctica* DSM100494, *Y. lutea* DSM19828, *Blastococcus* sp. DSM4427 and also *Terrabacter* sp. DSM102553 and *Terrabacter* sp. DSM102554 for the selected *Terrabacter* sp. were obtained from DSMZ.

### ***Determination of vitamin B***_***12***_*** and pseudovitamin B***_***12***_*** synthesis by the selected strains***

**Fig. 2:**
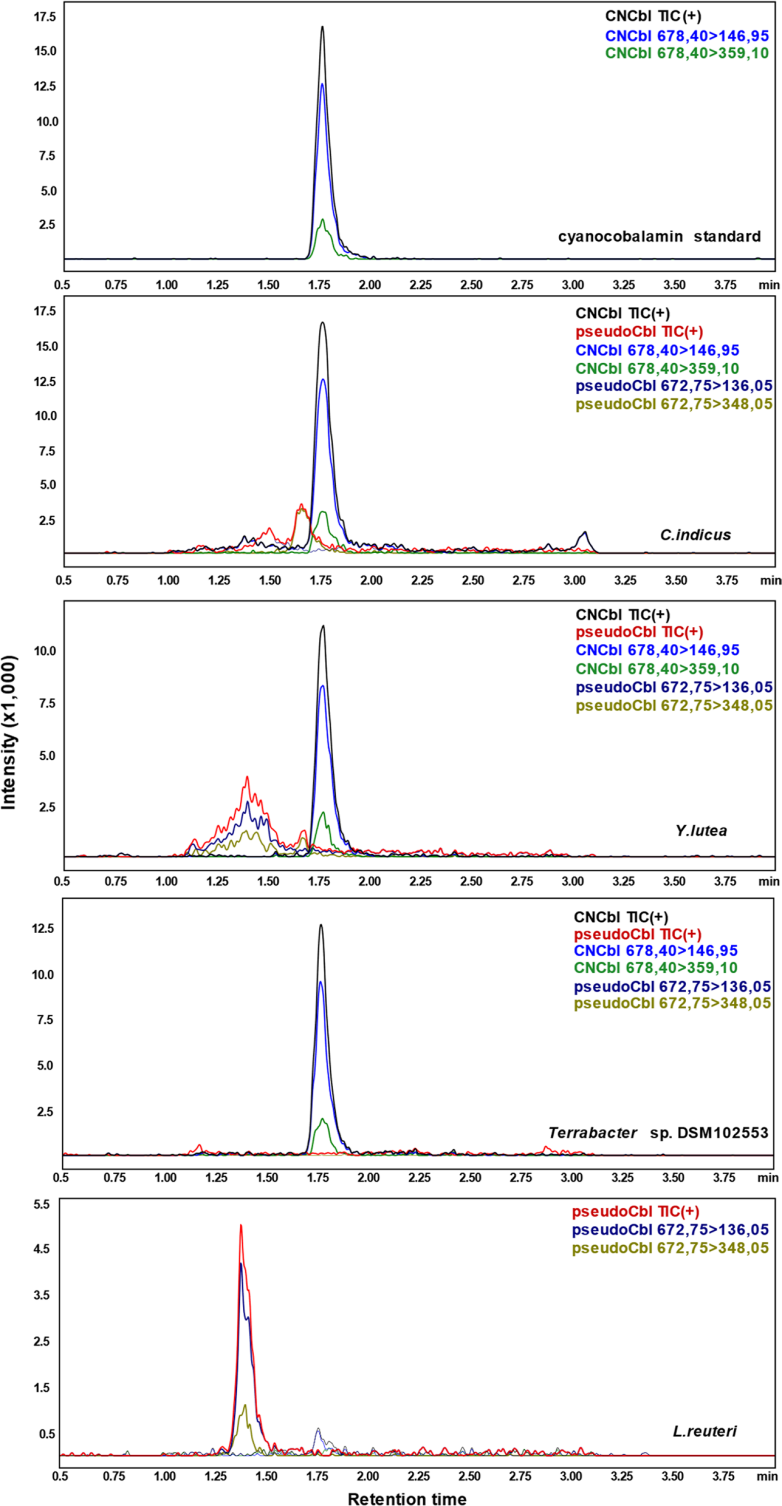
Identification of the active and inactive cobalamin forms in the cell extracts of the selected producing strains. Shown are TICs (total ion currents) and traces corresponding to the fragment ions originating from the identified parent mass obtained from the cyanocobalamin reference compound, cell extracts of the selected strains and of the pseudocobalamin produced by *L. reuteri*

To investigate the ability of the identified strains to produce cobalamins, the cell extracts were analyzed with the developed LC–MS/MS method and compared with cyanocobalamin and pseudocobalamin reference compounds. Since various microorganisms can produce different B_12_ analogues, where DMBI is replaced by other benzimidazoles, purines or phenolic compounds (Crofts et al. 2013), we aimed to identify only those producing the active vitamin B_12_. As the active and inactive forms differ in their respective ligands (Fig. 1), their fragmentation patterns are characteristic and can be used do differentiate between the two forms. The spectrum of the cyanocobalamin reference compound demonstrated a parent ion with m/z 678.40 and fragment signals with m/z 146.95 [DMBI + H]^+^ and m/z 359.10 [DMBI + sugar + PO_3_ + H]^+^, while a parent ion with m/z 672.75 and fragment signals with m/z 136.05 [adenine + H]^+^ and m/z 348.05 [adenine + sugar + PO_3_ + H]^+^ were detected in the spectrum of the pseudovitamin B_12_ (Fig. 2). These results correlate with the data reported in previous studies (Chamlagain et al. 2017; Bernhardt et al. 2019).

Among the selected strains, cobalamins were detected in the extracts of *Y. lutea, C. indicus* and *Terrabacter* sp. DSM102553 (Fig. 2), while LC–MS/MS analysis of *Terrabacter* sp. DSM102554, *R. antarctica* DSM100494 and *Blastococcus* sp. DSM44272 revealed no production of the active cobalamin (Supplementary Fig. 2). Nevertheless, further investigations can be performed to analyze, whether the mentioned microorganisms can produce vitamin B_12_ under certain other conditions or in other cultivation media. For example, supplementation with FMNH_2_ necessary for the formation of DMBI (Deptula et al. 2015) might facilitate synthesis of the vitamin B_12_. The lack of cobalamin production in *Terrabacter* sp. DSM102554, *R. antarctica* DSM100494 and *Blastococcus* sp. DSM44272 might be due to the presence of an incomplete biosynthetic way or to transcriptional repression of vitamin B_12_ synthesis gene expression. In future research, these strains can be examined for the presence of the other genes of the vitamin B_12_ synthesis pathway and the expression levels of the corresponding genes can be analyzed.

In the case of the three producing strains, the active form was prevalent in their cell extracts, since the characteristic retention time and fragmentation pattern observed for the cyanocobalamin standard were also detected in the cell extracts of these examined candidates. Moreover, the retention time and fragmentation pattern of the second minor peak identified in the chromatogram of *Y. lutea* demonstrated production of low amounts of pseudovitamin B_12_. Additionally, an unidentified peak was detected in the chromatogram of *C. indicus,* while no other compounds except the active vitamin B_12_ were detected for *Terrabacter* sp. DSM102553.

### ***Growth comparison of vitamin B***_***12***_***-producing strains in complex media***

**Fig. 3:**
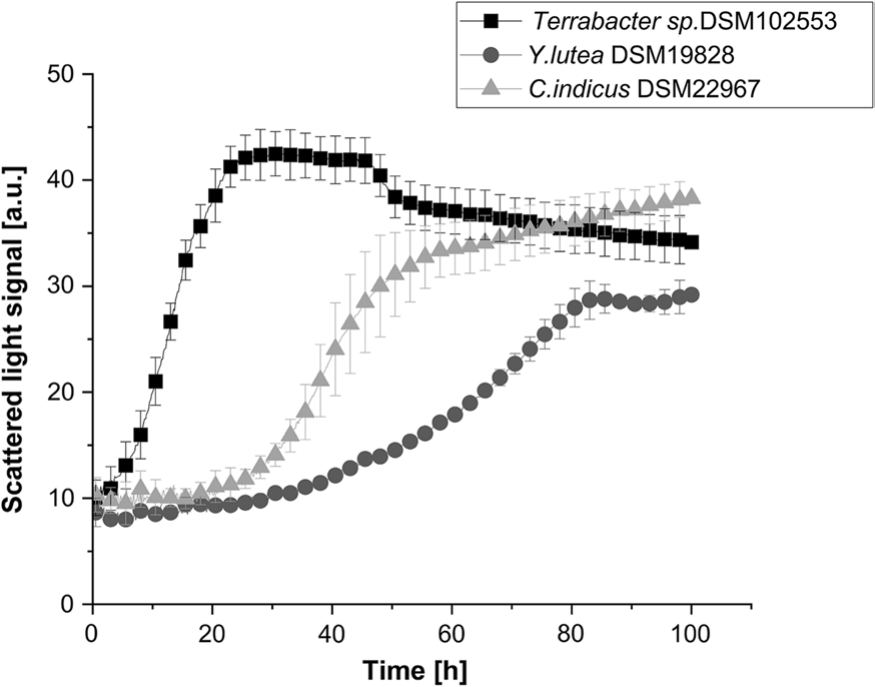
Comparison of *Y. lutea, C. indicus* and *Terrabacter sp.* DSM102553 growth curves. Respective complex media recommended by DSMZ were used (medium 65 for *Y.* *lutea*, medium 92 for *C. indicus*, medium 513 for *Terrabacter sp*. DSM102553). The data points represent the mean values and standard deviations of three biological replicates

To get a first impression of the growth behavior of the vitamin B_12_-producing strains, their growth curves in respective complex media recommended by DSMZ were determined. As Fig. 3 shows, remarkable differences in the growth behaviour of the investigated strains could be observed. The cultures of *C. indicus* demonstrated a lag-phase of approx. 20 h, which was followed by the exponential growth phase after which a linear biomass accumulation continued. The growth of *Y. lutea* under the tested conditions was characterized by a prolonged lag-phase if compared to *C. indicus* and *Terrabacter* sp. DSM102553 and the maximum scattered light signal of approx. 25 a. u. after 80 h. Among three tested strains, *Terrabacter* sp. DSM102553 demonstrated the highest maximum scattered light signal of 42 a. u. already after 25 h of cultivation.

Since the same media were applied for the precultures and main cultivation experiment, which was started at the same OD_600_ for all strains, these differences cannot be explained by the adaptation of the cells to growth in new cultivation medium but are rather due to characteristic features of each specific strain. Therefore, the above demonstrated ability to accumulate higher biomass during a shorter time period indicates that *Terrabacter* sp. DSM102553 is the most appropriate strain for vitamin B_12_ production among the identified candidates under the tested conditions.

Moreover, since no pseudovitamin B_12_ or unidentified peaks were detected in the chromatograms of *Terrabacter* sp. DSM102553 extracts (Fig. 2), the strain was selected for further investigation.

### ***Production of active vitamin B***_***12***_*** with Terrabacter sp. DSM102553 in different media***

**Fig. 4:**
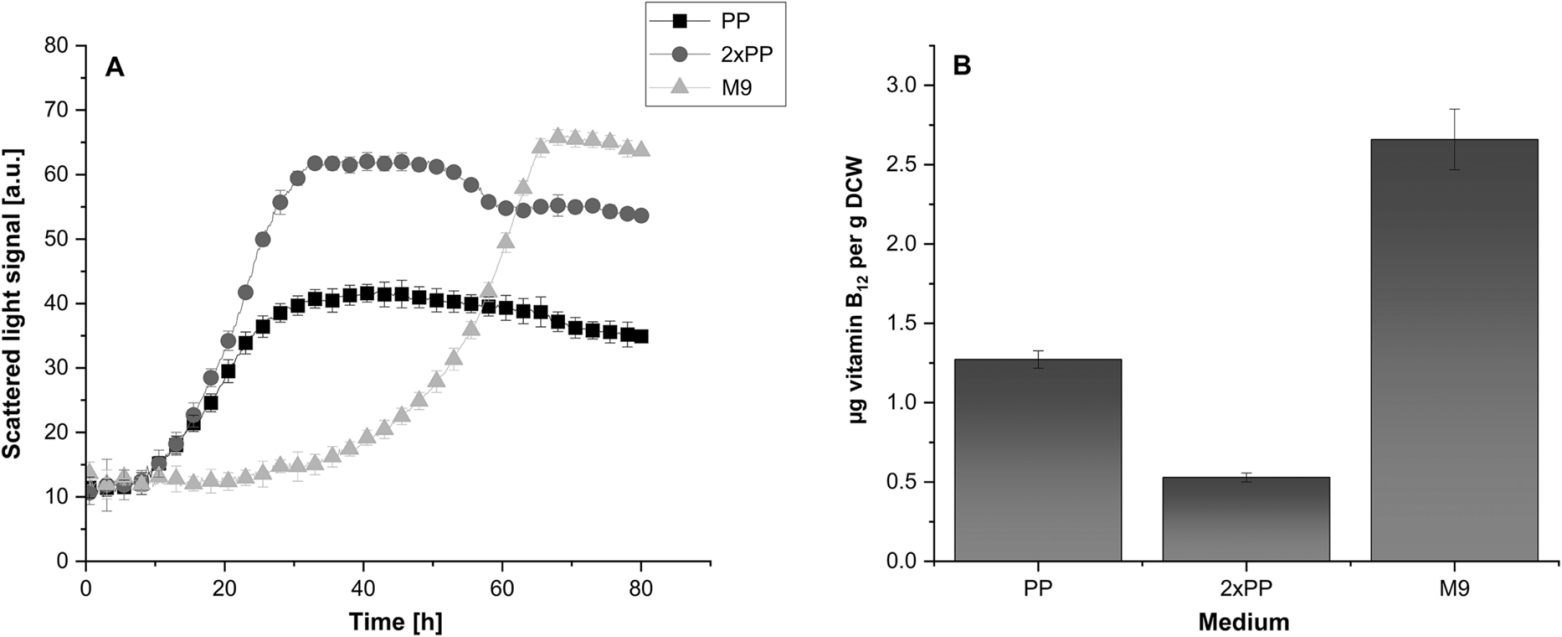
Growth and vitamin B_12_ production capability of *Terrabacter sp.* DSM102553 in different media **A** Growth curves of *Terrabacter sp.* DSM102553 in PP, 2xPP and M9 medium. **B** Vitamin B_12_ content in cells cultured in PP, 2xPP and M9 medium. The data points represent the mean values and standard deviations of three biological replicates

In order to improve cell density and thereby volumetric vitamin B_12_ productivity of *Terrabacter sp*. DSM102553, we tested two-fold concentrated PP medium. However, as peptones are one of the most expensive components of microbial media, we aimed to find a suitable cheap minimal medium. For this purpose, precultures grown in the standard PP medium were inoculated in PP, 2xPP and M9 medium and the growth of *Terrabacter* sp. DSM102553 was compared. As shown in Fig. 4a, the maximal cell density in 2xPP and M9 medium was about 1.5 times higher in comparison to PP. Although a prolonged lag phase was observed in M9 medium, the maximum cell density in M9 medium was comparable with that reached in 2xPP. Our further investigations have also shown that biotin and thiamin have no influence on the growth of the strain (Supplementary Fig. 3), which is why they were excluded from the medium composition in the vitamin B_12_ quantification experiments.

The analysis of vitamin B_12_ production was performed in PP, 2xPP and M9 medium after 100 h of cultivation as higher amounts of produced cobalamin at later growth stages have already been reported for *P. freudenreichii* (Deptula et al. 2017). Among the three media, the highest vitamin B_12_ production level was observed in M9 medium (Fig. 4b). The amounts of 2.65 µg of vitamin B_12_ per g dry cell weight (DCW) obtained with this medium were two-fold and five-fold higher than those produced in PP or 2xPP medium, respectively. In comparison to PP medium, faster growth and higher cell density, but lower vitamin B_12_ concentrations were observed in 2xPP medium, which shows that under these conditions the production of vitamin B_12_ was not linked to biomass formation in *Terrabacter sp.* DSM102553.

By using the presence of *bluB*/*cobT2* fusion genes as a potential indicator for efficient production of active vitamin B_12_, we were able to identify strains which produce vitamin B_12_ under aerobic conditions. Since oxygen is necessary for DMBI formation and active vitamin B_12_ synthesis in *P. freudenreichii* (Deptula et al. 2015), a two-step cultivation including aerobic and anaerobic stages is applied for vitamin B_12_ production with these microaerophilic strains (Chamlagain et al. 2015, 2017; Deptula et al. 2017). The omission of the anaerobic cultivation step can be an attractive advantage since it makes the synthesis procedure more easy-to-handle and time-effective. So far, the vitamin B_12_ contents reported in this manuscript cannot compete with the values of approx. 6–15 µg per g wet cell mass reported for *P. freudenreichii* (Deptula et al. 2017) in a comparable cultivation in shaking flasks, but the achieved levels obtained with minimal medium are promising. Future optimization steps can involve further medium optimization, for example via supplementation of DMBI (Chamlagain et al. 2016) or cobalt (Mohammed et al. 2014) as well as identification of mutants with higher productivity.

## Conclusions

Our strategy for the identification of new vitamin B_12_ producing microorganisms resulted in the description of bacteria with the ability to produce active vitamin B_12_ under aerobic conditions. Cobalamin analysis was performed with a sensitive optimized LC–MS/MS method with a wide concentration range developed in this study which not only allows vitamin B_12_ quantification, but also enables clear discrimination between its active and inactive form and simplifies selective screening processes for potential producers. The identified ability of *Terrabacter* sp. DSM102553 to synthesize active cobalamin in the minimal medium demonstrated in our work opens new opportunities for further optimization of the newly identified strain and its possible application for biotechnological vitamin B_12_ synthesis in a fairly simple aerobic manner.

## Supplementary Information

Below is the link to the electronic supplementary material.

Supplementary file1 (DOCX 3362 KB)

Supplementary file2 (XLSX 10 KB)

Supplementary file3 (XLSX 98 KB)

## Data Availability

The data generated during the is available in the manuscript and in the Supplementary Information.

## References

[CR1] Bernhardt C, Zhu X, Schütz D, Fischer M, Bisping B (2019). Cobalamin is produced by *Acetobacter pasteurianus* DSM 3509. Appl Microbiol Biotechnol.

[CR2] Campos-Gimnez E, Fontannaz P, Trisconi M-J, Kilinc T, Gimenez C, Andrieux P (2008). Determination of Vitamin B_12_ in food products by liquid chromatography/UV detection with immunoaffinity extraction: single-laboratory validation. J AOAC Int.

[CR3] Chamlagain B, Edelmann M, Kariuloto S, Ollilainen V, Piironen V (2015). Ultra-high performance liquid chromatographic and mass spectrometric analysis of active vitamin B_12_ in cells of *Propionibacterium* and fermented cereal matrices. Food Chem.

[CR4] Chamlagain B, Deptula P, Edelmann M, Kariluoto S, Grattepanche F, Lacroix C, Varmanen P, Piironen V (2016). Effect of the lower ligand precursors on vitamin B_12_ production by food-grade *Propionibacteria*. LWT—Food Sci Technol.

[CR5] Chamlagain B, Sugito T, Deptula P, Edelmann M, Kariuloto S, Varmanen P, Piironen V (2017). In situ production of active vitamin B_12_ in cereal matrices using *Propionibacterium freudenreichii*. Food Sci Nutr.

[CR6] Crofts T, Seth E, Hazra A, Taga M (2013). Cobamide structure depends on both lower ligand availability and CobT substrate specificity. Chem Biol.

[CR7] Deptula P, Kylli P, Chamlagain B, Holm L, Kostiainen R, Piironen V, Savijoki K, Varmanen P (2015). BluB/CobT2 fusion enzyme activity reveals mechanisms responsible for production of active form of vitamin B_12_ by *Propionibacterium freudenreichii*. Microb Cell Fact.

[CR8] Deptula P, Chamlagain B, Edelmann M, Sangsuwan P, Nyman T, Savijoki K, Piironen V, Varmanen P (2017). Food-Like Growth Conditions Support Production of Active Vitamin B_12_ by *Propionibacterium freudenreichii* 2067 without DMBI, the Lower Ligand Base, or Cobalt Supplementation. Front Microbiol.

[CR9] Kummer MJ, Lee YS, Yuan M, Alkotaini B, Zhao J, Blumenthal E, Minteer SD (2021). Substrate channeling by a rationally designed fusion protein in a biocatalytic cascade. JACS Au.

[CR10] Lee J, Shin J, Park J, Kim H, Ahn J, Kwak B, Kim J (2015). Analytical determination of Vitamin B_12_ content in infant and toddler milk formulas by liquid chromatography tandem mass spectrometry (LC-MS/MS). Korean J Food Sci Anim Resour.

[CR11] Lu B, Ren Y, Huang B, Liao W, Cai Z, Tie X (2008). Simultaneous determination of four water-soluble vitamins in fortified infant foods by ultra-performance liquid chromatography coupled with triple quadrupole mass spectrometry. J Chromatogr Sci.

[CR12] Luo X, Chen B, Ding L, Tang F, Yao S (2006). HPLC-ESI-MS analysis of Vitamin B_12_ in food products and in multivitamins-multimineral tablets. Anal Chim Acta.

[CR13] Martens J, Barg H, Warren M, Jahn D (2002). Microbial production of vitamin B_12_. Appl Microbiol Biotechnol.

[CR14] Mohammed Y, Lee B, Kang Z, Du G (2014). Development of a two-step cultivation strategy for the production of vitamin B_12_ by *Bacillus megaterium*. Microb Cell Fact.

[CR15] Perlman D (1959) Microbial Synthesis of Cobamides 1:87–122. doi: 10.1016/s0065-2164(08)70476-3 10.1016/s0065-2164(08)70476-3 13854292

[CR16] Rüdiger J, Schwab W (2019). Improving an *Escherichia coli*-based biocatalyst for terpenol glycosylation by variation of the expression system. J Ind Microbiol Biotechnol.

[CR17] Schwertner H, Valtier S, Bebarta V (2012). Liquid chromatographic mass spectrometric (LC/MS/MS) determination of plasma hydroxocobalamin and cyanocobalamin concentrations after hydroxocobalamin antidote treatment for cyanide poisoning. J Chromatogr B Analyt Technol Biomed Life Sci.

[CR18] Szterk A, Roszko M, Małek K, Czerwonka M, Waszkiewicz-Robak B (2012). Application of the SPE reversed phase HPLC/MS technique to determine vitamin B_12_ bio-active forms in beef. Meat Sci.

[CR19] Takahashi-Iñiguez T, García-Hernandez E, Arreguín-Espinosa R, Flores ME (2012). Role of vitamin B_12_ on methylmalonyl-CoA mutase activity. J Zhejiang Univ Sci B.

[CR20] van der Berg H, Daqnelie P, van Staweren W (1988). Vitamin B_12_ and seaweed. Lancet.

[CR21] Watanabe F (2007). Vitamin B_12_ sources and bioavailability. Exp Biol Med (maywood).

[CR22] Watanabe F, Katsura H, Takenaka S, Fujita T, Abe K, Tamura Y, Nakatsuka T, Nakano Y (1999). Pseudovitamin B_(12)_ is the predominant cobamide of an algal health food, spirulina tablets. J Agric Food Chem.

[CR23] Watanabe F, Miyamoto E, Nakano Y (2001). Inactive corrinoid-compound significantly decreases in *Spirulina platensis* grown in a cobalt-deficient medium. J Agric Food Chem.

[CR24] Weyden MVD, Cooper M, Firkin B (1973). Defective DNA synthesis in human megaloblastic bone marrow: effects of hydroxy-B_12_ 5′-deoxyadenosyl-B_12_ and methyl-B_12_. Blood.

[CR25] Yi S, Seth EC, Men Y-J, Stabler SP, Allen RH, Alvarez-Cohen L, Taga ME (2012). Versatility in corrinoid salvaging and remodeling pathways supports corrinoid-dependent metabolism in *Dehalococcoides mccartyi*. Appl Environ Microbiol.

[CR26] Zironi E, Gazzotti T, Barbarossa A, Farabegoli F, Serraino A, Pagliuca G (2014). Determination of Vitamin B_12_ in dairy products by ultra performance liquid chromatography-tandem mass spectrometry. Ital J Food Saf.

