# Supplementary material for: Identification of vitamin B12 producing bacteria based on the presence of bluB/cobT2 homologues
Source: Biotechnol Lett. 2023 Mar 13;45(4):563–72. doi: 10.1007/s10529-023-03362-2 (PMC10038948; doi:10.1007/s10529-023-03362-2)
Supplement: Supplementary file 1 — Supplementary file1 (DOCX 3362 KB) [file 10529_2023_3362_MOESM1_ESM.docx]

**Supporting Information**

**Supplementary Figure 1** Selectivity of the method to cyanocobalamin in the presence of the acetate buffer and cell matrix. Figure shows the chromatograms corresponding to (A) buffer blank, (B) buffer spiked with CNCbl standard, (C) cell matrix blank obtained from the non-producing strain *E. coli* DSM18039 and (D) cell matrix spiked with CNCbl standard.

**Supplementary Figure 2** LC-MS/MS chromatograms of the cyanocobalamin reference compound and the cell extracts of *R. antarctica* DSM100494, *Blastococcus* sp. DSM44272 and *Terrabacter* sp. DSM102554. Shown are representative LC-MS/MS chromatograms of the cyanocobalamin reference compound and of the cell extracts of the selected strains.

**Supplementary Figure 3** Growth curves of *Terrabacter* sp. DSM102553 in M9 medium with lowered concentrations of biotin and thiamin or without biotin and thiamin supplementation. The data points represent the mean values and standard deviations of three biological replicates.

**Supplementary Information**

Supplementary Figure 1


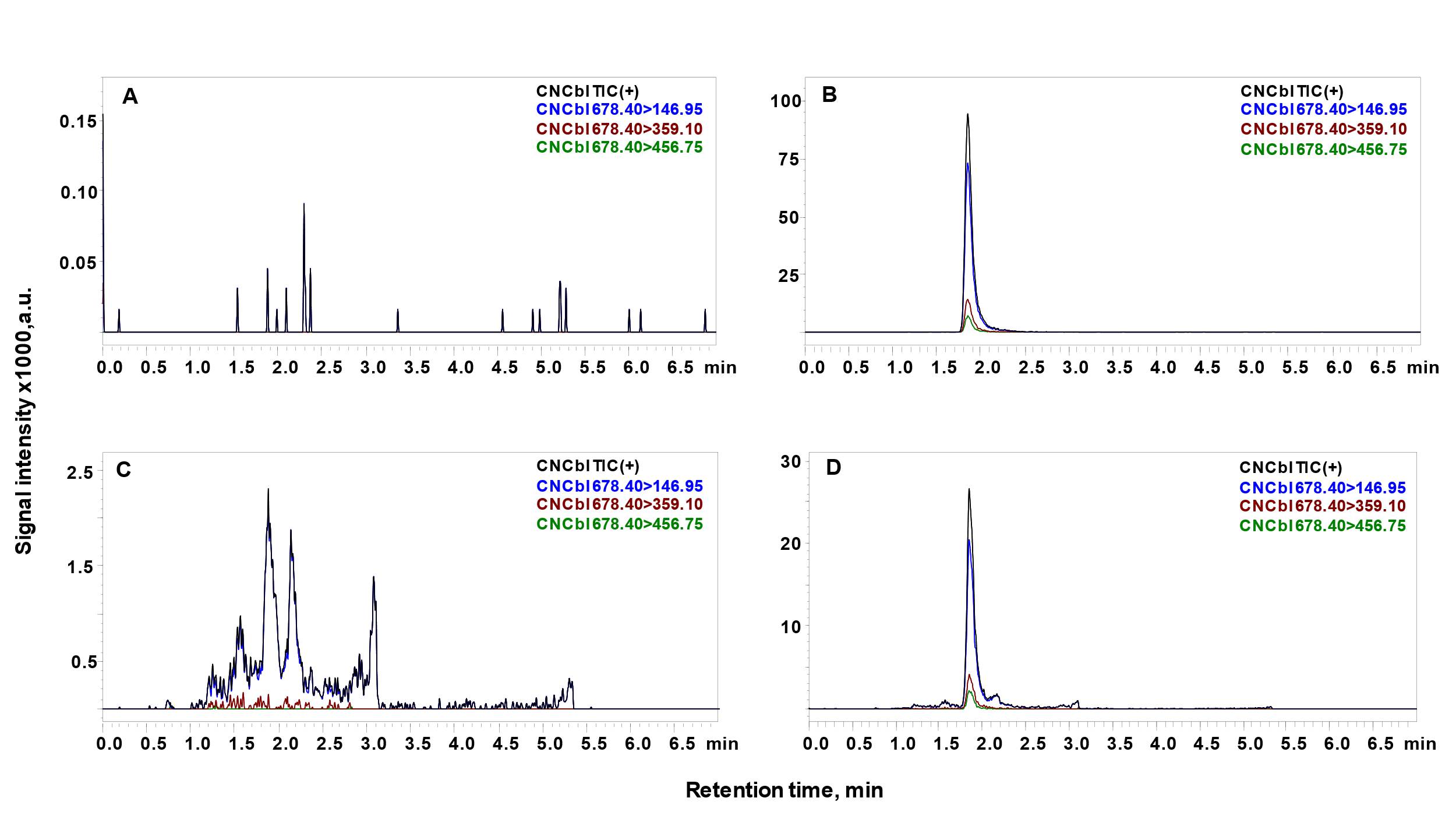


Supplementary Figure 2


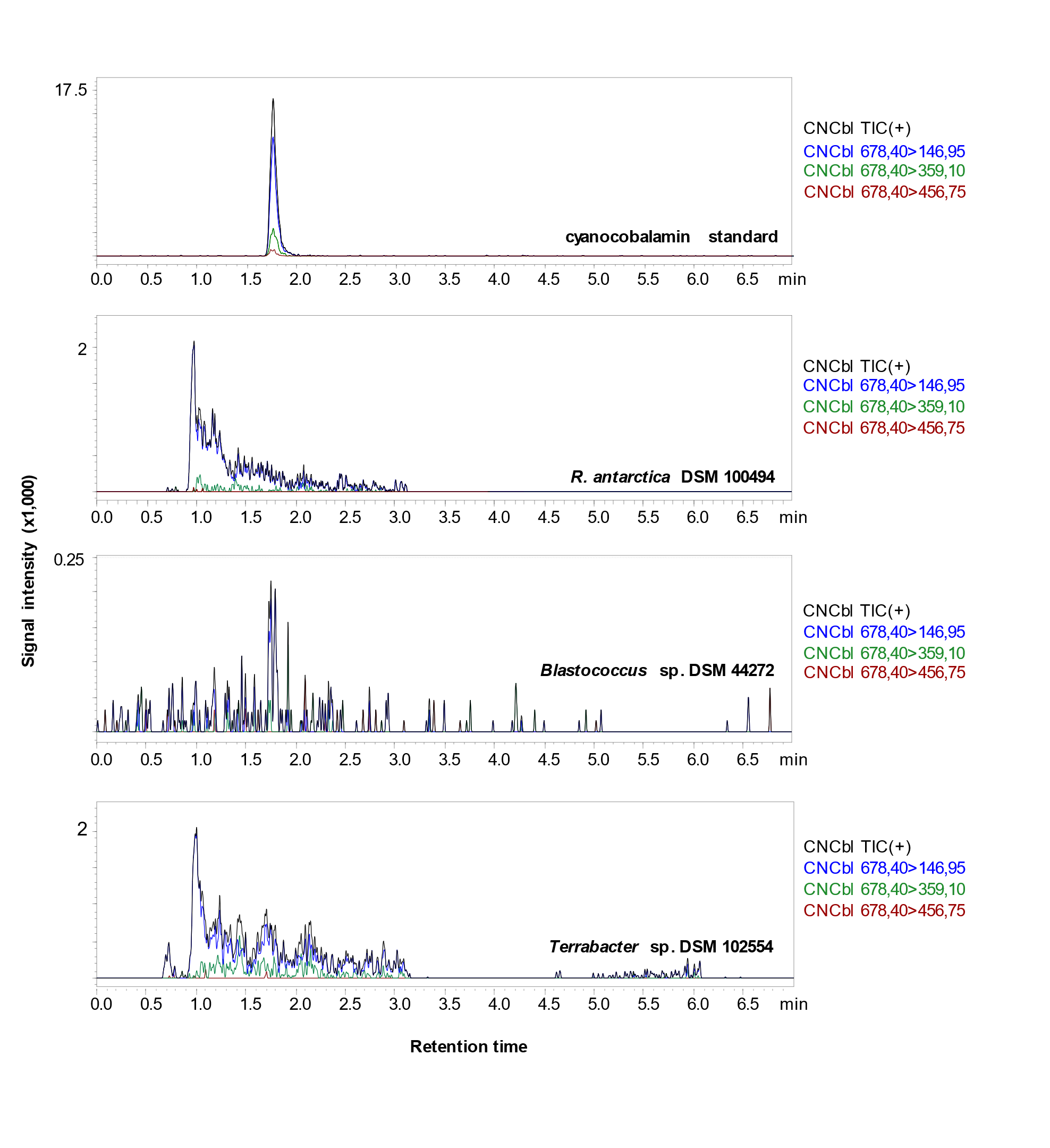


Supplementary Figure 3
